# Simultaneous Spatial Mapping and Identification of C-Peptides Alongside Lipids in Mouse Pancreatic Tissues with Nano-DESI Mass Spectrometry Imaging

**DOI:** 10.1016/j.mcpro.2026.101632

**Published:** 2026-07-31

**Authors:** Manxi Yang, Mushfeqa Iqfath, Frederick Nguele Meke, Zihan Qu, Emerson Hernly, Xindi Tang, Pei Su, Zhong-Yin Zhang, Julia Laskin

**Affiliations:** 1 Department of Chemistry, Purdue University, West Lafayette, Indiana, USA; 2 Borch Department of Medicinal Chemistry and Molecular Pharmacology, Purdue University, West Lafayette, Indiana, USA; 3 Department of Chemistry, Northwestern University, Evanston, Illinois, USA; 4 James Tarpo Jr. and Margaret Tarpo Department of Chemistry, Purdue University, West Lafayette, Indiana, USA

**Keywords:** peptides, islets of Langerhans, mass spectrometry imaging (MSI), nanospray desorption electrospray ionization (nano-DESI), immunofluorescence (IF) imaging, molecular networking

## Abstract

Peptides secreted by pancreatic islet cells regulate glucose homeostasis. Their dysregulation may contribute to metabolic disorders such as diabetes and pancreatic disease. Here, we use nanospray desorption electrospray ionization mass spectrometry imaging (nano-DESI MSI) to identify and map intact and truncated C-peptides in mouse pancreatic tissue. Alongside commonly observed lipids and metabolites, we detected 36 multiply-charged acidic peptides that preferentially ionize in negative ion mode. On-tissue tandem mass spectrometry (MS/MS) was used to identify them as intact C-peptide isoforms and multiple truncated variants, including several previously unreported peptides. Molecular networking of MS/MS data assigned some unidentified species to C-peptide families based on shared fragmentation patterns. Ion images revealed distinct distributions of truncated peptides, with chemical gradients extending from β-cells into peri-insular acinar cells, suggesting their potential roles in endocrine–exocrine communication. These findings demonstrate the power of nano-DESI MSI to identify and spatially map novel peptides, which is critical for understanding their roles in pancreatic tissues.

## Highlights

• First insights into C-peptide diversity and roles in pancreatic and islet biology.
• Nano-DESI MSI enables spatial mapping of intact and truncated C-peptides.
• Identification of both previously reported and novel truncated C-peptides using on-tissue MS/MS and molecular networking.
• 36 multiply charged peptides exhibit distinct localization patterns, suggesting their role in endocrine–exocrine communication.

## Graphical Abstract

**Figure:**
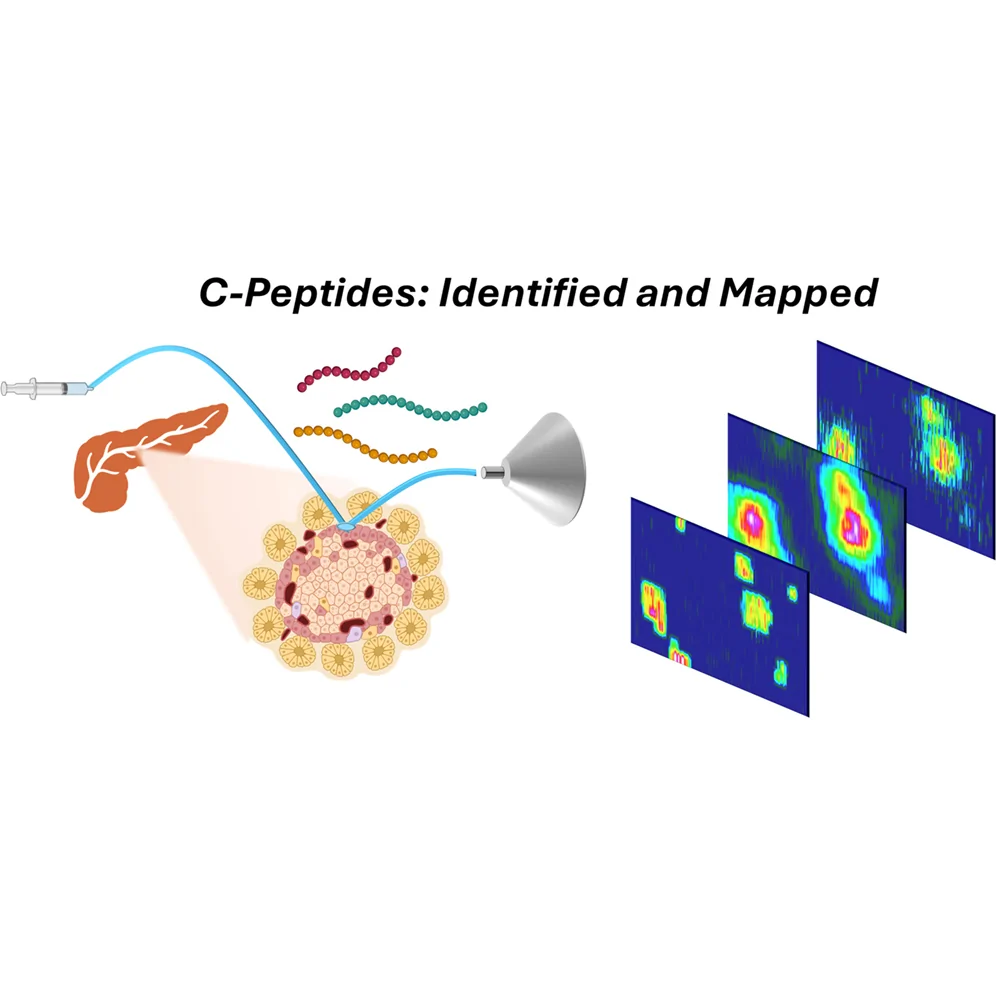

## In Brief

Despite growing interest in understanding the role of C-peptides in pancreatic biology, their molecular diversity and spatial localization remain poorly characterized. This study used nano-DESI mass spectrometry imaging to identify and spatially map intact and truncated C-peptides directly in mouse pancreatic tissue. On-tissue MS/MS and molecular networking helped define their identities and structural relationships. Distinct localization patterns and chemical gradients extending from β-cells into peri-insular acinar cells suggest potential roles for these peptides in pancreatic biology and endocrine-exocrine communication.

The pancreatic islets of Langerhans, comprising only ∼2% of the pancreas, are critical for regulating blood glucose levels (1). Different cell types in the islets contribute to the delicate balance of hormones involved in glucose metabolism and overall metabolic regulation within the body, primarily through the secretion of insulin by β-cells and glucagon by α-cells (2). Insulin is synthesized as a preprohormone and subsequently processed to proinsulin, which undergoes proteolytic cleavage to generate mature insulin and its equimolar byproduct, C-peptide (3). Upon cleavage, bioactive insulin and free C-peptide are released. Once considered an inert marker of insulin production, C-peptide is now recognized as a bioactive peptide that modulates vascular and peripheral nerve function, with anti-inflammatory and cytoprotective effects in diabetes (4). In clinical studies, the C-peptide test has been used to differentiate type 1 from type 2 diabetes (5).

In addition to intact C-peptide 1 and 2, several C-peptide variants generated via enzymatic truncation within β-cell secretory granules or variation in prohormone processing in pancreatic islets have been identified (6, 7). Despite the limited understanding of the role of specific C-peptide variants in pancreatic biology, growing evidence implicates C-peptide dysregulation in diabetes and some pancreatic diseases (8). For example, truncated C-peptides proteolytically cleaved at positions 21, 24, 26, and 30 have been identified in human insulinoma cells, indicating that C-peptide, but not insulin, undergoes proteolytic degradation in β-cells (8). In rodents, two C-peptide isoforms, C-peptide 1 and C-peptide 2, are present in the pancreas, and their intact forms are biologically active hormones with functions distinct from those of insulin (9, 10). In mice, C-terminally truncated C-peptides, including des-(25–29)-C-peptide 1 and des-(27–31)-C-peptide 2, each lacking five C-terminal residues, have been detected in pancreatic tissue extracts (11), with additional truncated variants reported in rat and mouse islets (6, 12, 13).

Despite their potential implications in islet biology and disease pathogenesis, little is known about the spatial localization of C-peptides within pancreatic tissues due to the small size of the islets and limited chemical specificity of optical microscopy methods. Mass spectrometry imaging (MSI) addresses this limitation as a label-free, highly sensitive method for multiplexed spatial mapping of metabolites, lipids, and proteins in tissue sections (14, 15, 16, 17), including the pancreas (18, 19, 20). Given the small size of pancreatic islets, combining MSI with microscopy techniques is a powerful strategy for enhancing spatial resolution, expanding molecular coverage, and contextualizing MSI molecular maps within tissue anatomy (21, 22, 23).

Previous MALDI MSI studies have established the feasibility of detecting pancreatic peptide hormones in tissues. Minerva *et al*. reported a strategy for improving peptide identification in MALDI MSI by combining high-mass-resolution profiling of an adjacent tissue section with bulk LC-MS/MS analysis of tissue extracts (24). Using this approach, they identified multiple insulin-derived C-peptides and evaluated their relative abundance in pancreatic islets. More recently, Sisnande *et al*. introduced an optimized MALDI MSI protocol for the peptidomic profiling of delipidated pancreatic tissue sections and islet extracts (13). While they successfully identified major pancreatic hormones and their proteoforms, representative ion images were reported for only one intact and one truncated C-peptide at a spatial resolution of 50 μm.

In this study, we present a comprehensive spatial analysis of C-peptides in pancreatic tissue using nanospray desorption electrospray ionization (nano-DESI) MSI, demonstrating that individual intact and truncated forms exhibit distinct distributions across pancreatic islets and the surrounding exocrine tissue. Nano-DESI is an ambient ionization technique, in which analytes are extracted into a dynamic liquid bridge formed at the sample surface and ionized by electrospray ionization (25, 26). The ability to tailor the solvent composition to improve the extraction and ionization efficiency of different classes of molecules enhances the sensitivity and expands the molecular coverage of lipids, metabolites, small molecules, glycans (27, 28, 29) and intact proteoforms (30, 31). In contrast to prior positive-ion MALDI-MSI workflows, in which tissue washing was used to mitigate ion suppression by removing endogenous lipids, the present nano-DESI MSI experiments were performed directly on untreated pancreatic tissue sections. Because C-peptides are enriched in acidic residues, including aspartic and glutamic acid, and contain few or no strongly basic amino acid residues, they were detected as multiply deprotonated species in negative-mode nano-DESI MSI alongside commonly observed lipids and metabolites that typically suppress ionization of endogenous peptides and proteins.

Using on-tissue tandem mass spectrometry (MS/MS) and molecular networking analysis (32), we identified a family of truncated C-peptides, including previously unreported species, based on fragmentation similarity to known C-peptide variants. By integrating high-resolution nano-DESI MSI with immunofluorescence (IF) microscopy, we obtained spatial maps of these species within mouse pancreatic islets. IF microscopy provided the roadmap for islet localization within the tissue, revealing sequence-dependent chemical gradients of these truncated variants between the endocrine and exocrine cells. Additionally, simultaneous detection of endogenous lipids that preferentially localize to islet or exocrine regions provides chemical context for evaluating C-peptide gradients across the endocrine–exocrine interface. This integrated analytical framework expands the molecular and spatial coverage of pancreatic C-peptides and provides insight into their processing and chemical gradients within the pancreatic microenvironment. Collectively, these results advance our understanding of C-peptide heterogeneity and spatial organization in the pancreas, providing a foundation for future investigations into the roles of truncated C-peptides in both health and disease.

## Experimental Procedures

### Chemicals and Materials

HPLC grade water and methanol (MeOH) were purchased from Fisher Chemical (Hampton, NH. Polymicro flexible fused silica capillary tubing (OD 150 μm, ID 50 μm; OD 360 μm, ID 100 μm; and OD 800 μm, ID 200 μm) was ordered from Molex (Thief River Falls, MN). CoraLite plus 488 INS mouse monoclonal antibody (Cat# CL488-66198) and CoraLite plus 647 glucagon rabbit polyclonal antibody (Cat# CL647-15954) were purchased from Proteintech (Rosemont, IL). DAPI (4ʹ,6-Diamidino-2-Phenylindole, Dihydrochloride) (Cat# Invitrogen D1306) was purchased from Thermo Fisher Scientific. Fluoromount was purchased from Diagnostic BioSystems Inc.

### Animal Tissue Preparation

Pancreatic tissues from 3- to 4-month-old male C57BL/6 mice were collected under an approved Purdue University IACUC protocol (#1511001324). After euthanasia, pancreata were excised, snap-frozen in chilled isopentane and cryosectioned at −21 °C into 18-μm slices using a CM1860 Cryostat. Sections were thaw-mounted onto positively charged slides and kept frozen until staining and imaging.

### Immunofluorescence Microscopy

Sections were fixed for 10 min in 4% paraformaldehyde in phosphate-buffered saline (PBS) and quenched with 100 mm glycine. A blocking buffer containing 5% goat serum, 2% BSA, 0.1% Triton X-100 and 0.1% sodium azide in PBS was applied for 1 h at room temperature. Slides were incubated overnight at 4 °C with CoraLite Plus 488 anti-insulin and CoraLite Plus 647 anti-glucagon antibodies (1:500 dilution). After washing with PBS, nuclei were stained with DAPI (1:1000 in PBS) for 10 min. Sections were mounted with Fluoromount and imaged on a Nikon A1Rsi confocal microscope (Nikon, Tokyo, Japan). Insulin, glucagon and nuclei were visualized using green (excitation: 488 nm, emission: 500–550 nm), far-red (excitation: 640 nm, emission: 663–738 nm), and blue (excitation: 405 nm, emission: 425–475 nm) channels, respectively.

### Nano-DESI MSI

Nano-DESI MSI experiments were performed on a Q-Exactive HF-X Orbitrap mass spectrometer (Thermo Fisher Scientific, Waltham, MA) equipped with a custom-designed nano-DESI source described in our previous publications (25, 26). Briefly, the probe consisted of two pulled fused-silica capillaries (∼10 μm tips) positioned at ∼90° to form a liquid bridge at the tissue surface. 9:1 methanol/water (v/v) solvent delivered at ∼0.5 μl/min was used to extract analytes while scanning the sample beneath the probe at 15 μm/s using a motorized stage. Spectra were recorded alternately in positive and negative ionization modes, with a line spacing of 30 μm. Spray voltages of +4.5 kV and −3.5 kV were applied, and data were collected over *m/z* 133 to 1700 at 60,000 resolving power (m/Δm at *m/z* 200); the AGC target was 1 × 10^6^; the maximum injection time was 200 ms. The multimodal IF and nano-DESI MSI workflow is illustrated in supplemental Fig. S1.

Positive-ion nano-DESI MSI of delipidated pancreatic tissue sections enabled detection of insulin proteoforms and intact glucagon; however, this workflow requires lipid removal and is not discussed in detail here. The present study focuses on direct analysis of untreated pancreatic tissue sections, which preserves endogenous lipids and metabolites and enables their simultaneous detection alongside peptides in the same experiment. Under these conditions, abundant lipids are retained and are likely to suppress many low-abundance peptide species. No truncated peptides derived from glucagon, insulin, or IAPP were confidently assigned using the identification criteria applied in this study.

### Image Generation and Registration

Ion images were generated using a custom Python-based software (https://github.com/LabLaskin/MSIGen) (33). Signals were extracted using a 10 ppm mass window, normalized to total ion current and converted to intensity map images. For multimodal integration, MSI and immunofluorescence images were registered by selecting at least three corresponding landmarks in each pair and computing an affine transformation. Registered images were combined by assigning each modality to an RGB channel.

### Tandem Mass Spectrometry (MS/MS) Data Acquisition and Analysis

On-tissue MS/MS experiments were performed on the Q-Exactive HF-X Orbitrap mass spectrometer using higher-energy collisional dissociation (HCD). MS/MS spectra were acquired by scanning the nano-DESI probe across a region of interest (e.g. endocrine, exocrine, and blood vessel regions). Peptides detected in negative ionization mode were fragmented using normalized collision energies of 20, 25, and 30 arbitrary units. The mass resolution was 60,000, and the mass isolation window was 3.0 Da. The AGC target was 1 × 10^5^ and the mass range of *m/z* 133 to 1999 was used in both modes.

Peptide assignments were supported by accurate precursor mass measurements and on-tissue MS/MS fragmentation. MS/MS spectra were manually annotated by matching the accurate *m/z* of the precursor ions and *m/z* of fragments to the predicted *m/z* of b, c, y ions and the corresponding neutral loss fragments of candidate sequences. For annotation of MS/MS spectra, we note that only confidently assigned terminal fragments and their analogs produced by one H_2_O or NH_3_ loss are annotated and that *m/z* of peptides reported in this study correspond to the most abundant isotopic peaks. Detailed annotation information, including precursor *m/z*, charge state, sequence, assigned fragment ions, fragment charge states, theoretical and observed fragment *m/z* values, and mass errors, is provided in supplemental Data S1. Fragment ion assignments were made using a maximum mass tolerance of 0.008 Da. Only species supported by accurate precursor mass and on-tissue MS/MS evidence are assigned as C-peptides; the remaining multiply charged ions are reported as unidentified species.

### Molecular Networking with GNPS

A molecular network was generated using the classical molecular networking workflow to assist peptide identification based on structural similarity among observed species. Raw MS/MS files were uploaded to GNPS2 (https://gnps2.org), and networks were constructed using cosine similarity of fragmentation spectra with precursor and fragment ion mass tolerances set to 0.05 Da. Edges were retained only for spectral pairs with a cosine score ≥0.7 and at least six matched fragment ions. The resulting networks were visualized and analyzed in Cytoscape (https://cytoscape.org), where nodes represent precursor ions and edges denote MS/MS spectral similarity to evaluate relationships between species.

### Experimental Design and Statistical Rational

Pancreatic tissue sections from 3–4-month-old male C57BL/6 mice were analyzed by nano-DESI MSI under consistent experimental conditions. Multiple biological replicate sections (n = 3) were prepared and examined to assess the reproducibility of peptide detection and spatial localization patterns. Acquisition parameters were held constant across all measurements. Peptide identities were assigned using accurate mass measurements, on-tissue MS/MS fragmentation patterns, and molecular networking analysis. Because this study was designed for peptide discovery and spatial characterization rather than quantitative comparison between experimental groups, ion images were normalized to total ion current, and no formal statistical hypothesis testing was performed.

## Results

By combining nano-DESI MSI of mouse pancreatic tissue sections with IF microscopy of insulin and glucagon for the localization of β- and α-cells in islets, respectively, we identified several lipids that can be used to localize pancreatic islets without IF imaging. We then used the ion images for studying spatial gradients of peptides secreted by endocrine cells into the surrounding exocrine tissue.

### Identifying Lipids Associated with Pancreatic Islets Using Nano-DESI MSI and IF Microscopy

**Fig. 1:**
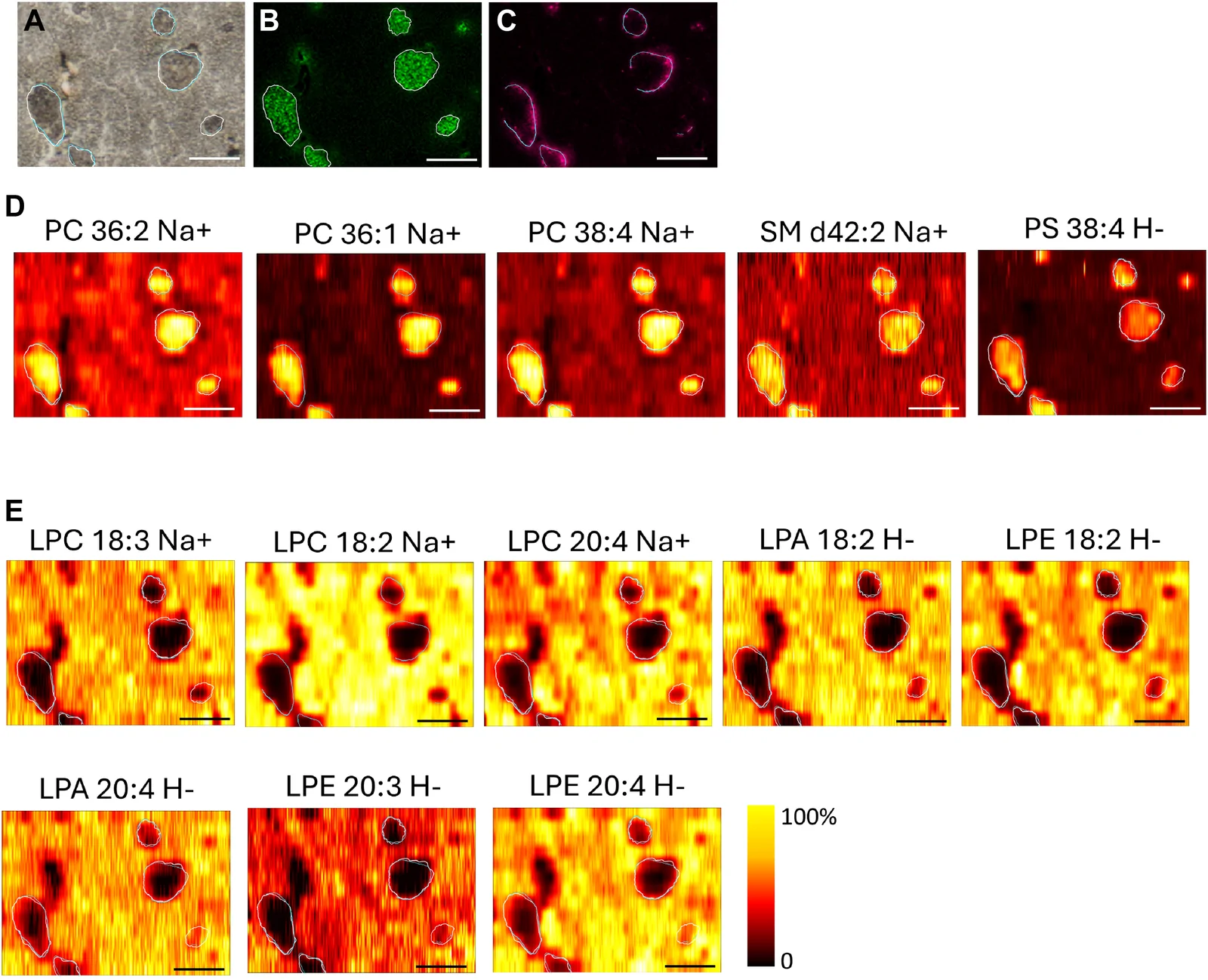
**Ion images of lipids and metabolites in mouse pancreas.** (*A*) Brightfield optical image of the analyzed region of the pancreatic tissue section. IF image of (*B*) insulin and (*C*) glucagon on the adjacent section. Nano-DESI ion images of lipids and metabolites normalized to TIC with (*D*) species showing enhanced abundance and (*E*) species showing diminished abundance in the islets. Scale bar: 250 μm. White trace delineates the location of endocrine cells based on the IF image of insulin in panel (*B*); *light blue* trace delineates the location of α-cells based on the IF image of glucagon in panel (*C*).

We previously demonstrated that several lipids are either tightly localized to pancreatic islets or show no signals in islets (20). In this study, we used dual-mode nano-DESI MSI of pancreatic tissues and IF microscopy of adjacent tissue sections to unambiguously identify lipid markers of islets as shown in Figure 1. Figure 1*A* shows a brightfield optical image of the analyzed tissue region. According to IF images of insulin (Fig. 1*B*) and glucagon (Fig. 1*C*), and consistent with a previous report (34), β-cells are found in the islet core and α-cells at the periphery. Nano-DESI MSI revealed distinct spatial distributions of multiple lipid species. Ion images showed enhanced abundances of phosphatidylcholine (PC) 36:2, PC 36:1, PC 38:4, sphingomyelin (SM) d42:2, and phosphatidylserine (PS) 38:4 within islet regions (Fig. 1*D*). In contrast, several lysolipids, including lysophosphatidylcholine (LPC) 18:3, LPC 18:2, LPC 20:4, lysophosphatidic acid (LPA) 18:2, lysophosphatidylethanolamine (LPE) 18:2, LPA 20:4, LPE 20:3, and LPE 20:4, showed no or very low signal within islets (Fig. 1*E*). These results are consistent with our previous report (20).

To further assess spatial relationships, line profiles extracted from lipid ion images were compared with insulin and glucagon profiles from IF microscopy (supplemental Fig. S2). Ion images from replicate datasets are shown in supplemental Figs. S3 and S4. The distributions of PC 36:1 and PS 38:4 closely match insulin-rich regions. The localization of PC 36:1 and PS 38:4 to IF-defined islet regions indicates that these lipid species can serve as endogenous markers of islets, eliminating the need for IF imaging and significantly simplifying the experimental workflow while preserving accurate spatial identification of islets. Such lipid-based markers provide a practical advantage for MSI studies of pancreatic tissues, particularly when integrating multiple molecular classes or when IF staining is not feasible.

### Identification of Truncated Forms of Pancreatic Peptides

**Fig. 2:**
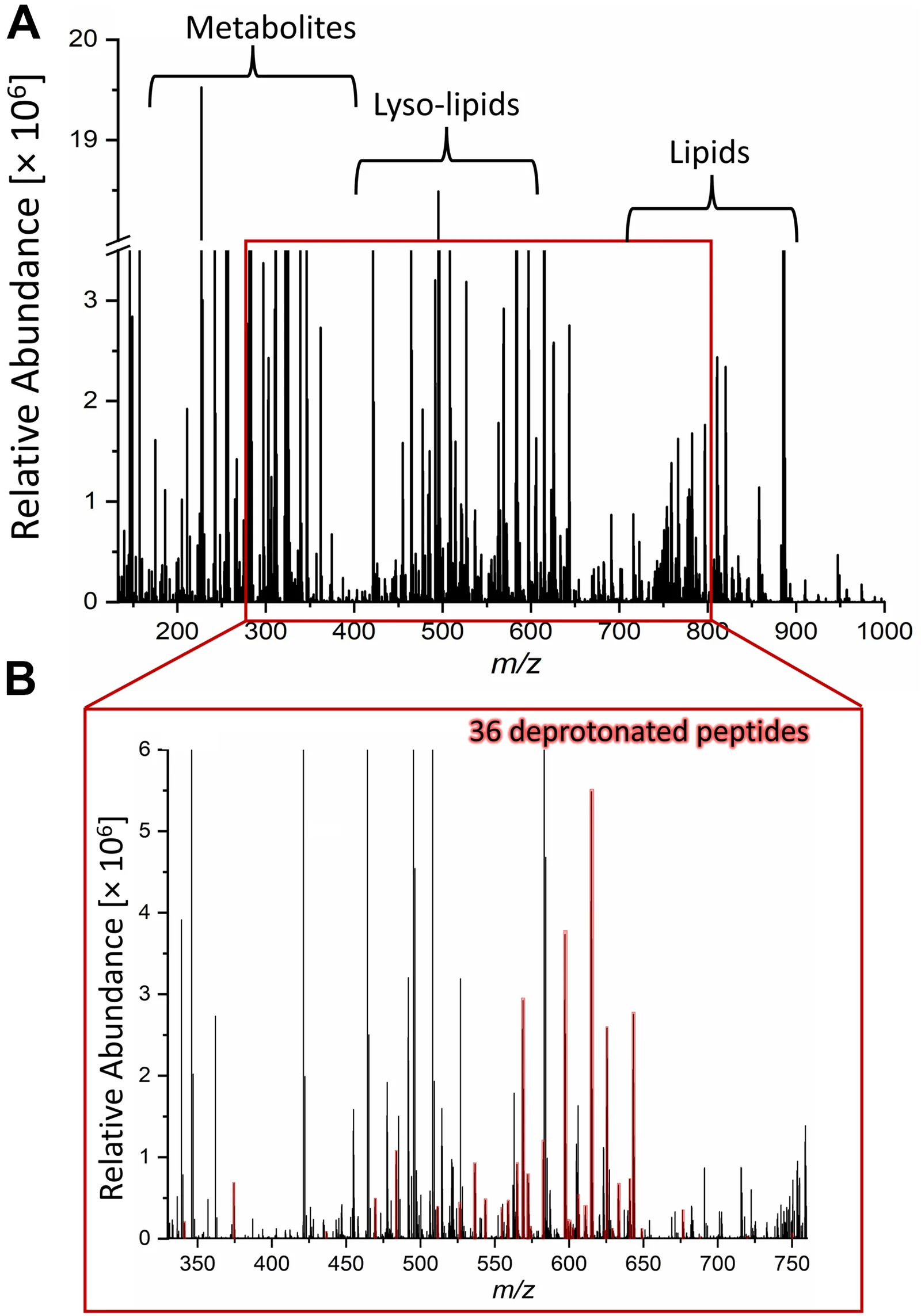
**Raw mass spectrum showing the simultaneous detection of lipids, metabolites, and peptides.** (*A*) Mass spectrum averaged over an islet and (*B*) zoomed-in spectrum highlighting peaks corresponding to deprotonated peptides.

Because of the efficient ionization of lipids in nano-DESI and other MSI modalities, peptides are often overlooked due to their lower signals. Figure 2 shows a mass spectrum averaged over one islet (Fig. 2*A*) highlighting the *m/z* regions in which metabolites, lysolipids, and lipids are observed. Unexpectedly, in negative-ion nano-DESI MSI of mouse pancreatic tissue sections performed without tissue washing, delipidation, matrix application, or other chemical pretreatment, we directly detected a family of intact and truncated C-peptides alongside these abundant lipid and metabolite signals. The expanded region of the mass spectrum shown in Figure 2*B* highlights 36 multiply charged species localized in and around the islets, including the C-peptide-related ions assigned in this study. These peptides were not detected in positive-mode nano-DESI MSI under the same sample preparation conditions, consistent with their acidic amino acid composition. C-peptides are enriched in acidic residues, including aspartic and glutamic acid, and contain few or no strongly basic amino acid residues, which facilitate their detection as multiply deprotonated species in negative mode. Thus, negative-ion nano-DESI MSI enabled simultaneous detection of lipids, metabolites, and C-peptides directly from pancreatic tissue sections while preserving the native chemical context of the tissue.

**Fig. 3:**
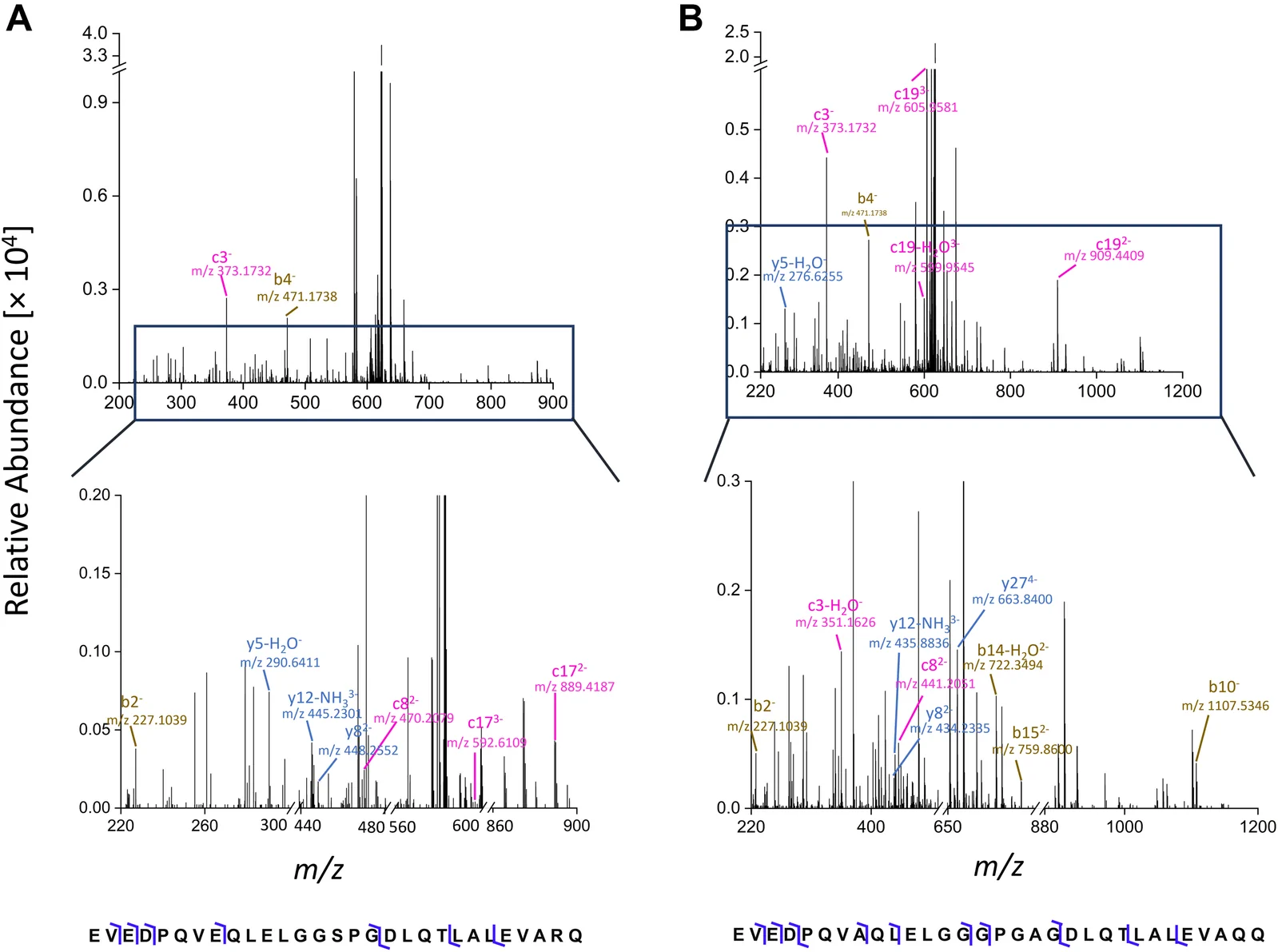
**On-tissue identification of the C-peptides by MS/MS.** MS/MS spectra (*top*), zoomed-in MS/MS spectra (*middle*), and fragmentation maps (*bottom*) of (*A*) −5 charge state of the 3121 Da C-peptide 1 and (*B*) −5 charge state of the 3133 Da C-peptide 2.

To identify these species, we acquired on-tissue MS/MS spectra as described in the Methods Section. Because negative ionization mode is rarely used in bottom-up proteomics, software for annotating peptide fragmentation spectra obtained in this mode is largely unavailable. Therefore, we manually annotated MS/MS spectra by proposing candidate peptide sequences, considering different isoforms and PTMs, and comparing predicted fragments with the experimental data. Using this approach, we identified two intact C-peptide isoforms, C-peptide 1 and C-peptide 2. Additionally, we identified four truncated peptides derived from the loss of C-terminal residues from C-peptide 1 and 10 peptides derived from C-peptide 2. A summary of the intact and truncated C-peptides identified in this study is provided in Supplemental Table S1 including peptide name, sequence, precursor charge state, observed *m/z*, neutral mass, and supporting assignment information. No modified C-peptides were observed in the present dataset. Peptide assignments were based on accurate precursor mass measurements, on-tissue MS/MS fragmentation, and manual annotation of fragment ions; therefore, database-search identification scores are not applicable. The annotated MS/MS spectra and the fragmentation maps of the two intact C-peptide isoforms are shown in Figure 3; the corresponding spectra obtained for truncated C-peptides are shown in supplemental Figs. S5–S18. Most of the observed fragments correspond to negatively charged b, c, y ions, and their analogs produced by H_2_O and NH_3_ losses in their MS/MS spectra (35, 36, 37). We note that only confidently assigned terminal fragments and their analogs produced by one H_2_O or NH_3_ loss are annotated in MS/MS spectra. Internal fragments and fragments corresponding to losses of other neutral molecules or multiple neutral losses are not labeled in the spectra.

### Molecular Networks Based on MS/MS Fragmentation Patterns

**Fig. 4:**
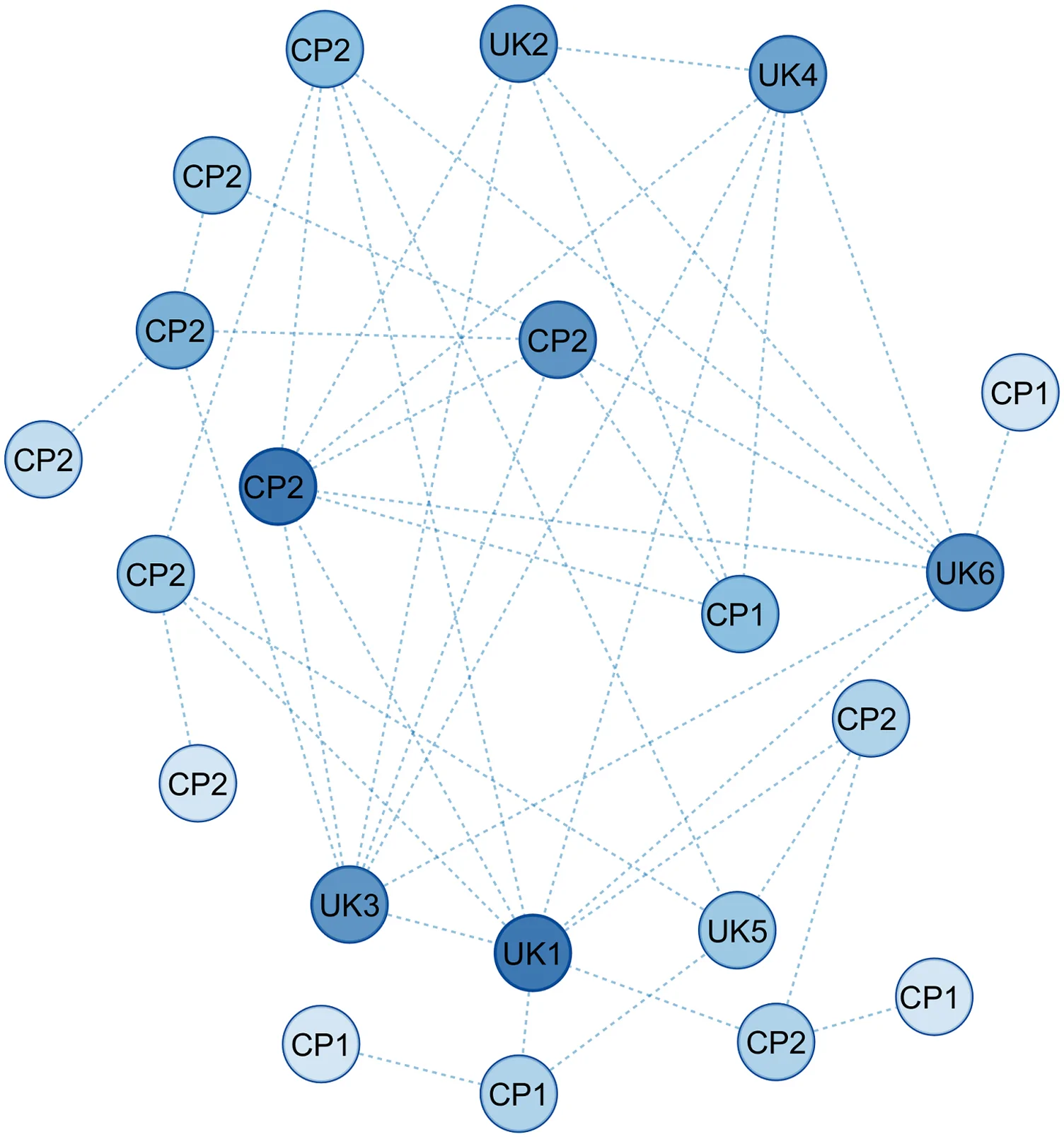
**A subset of the GNPS molecular network showing consensus spectra clustered by spectral similarity.** Nodes represent consensus MS/MS spectra and edges indicate cosine similarity above the defined threshold. Node labels correspond to intact or truncated C-peptide 1 (CP1), C-peptide 2 (CP2), and unidentified species (UK).

The manual annotation of MS/MS spectra allowed us to identify 16 C-peptide species, with 20 additional peptide species remaining unidentified. To aid peptide identification by leveraging structural similarity among detected species, we employed GNPS classical molecular networking (32). A representative subnetwork obtained using the parameters listed in the Supplemental Information is shown in Figure 4, where the nodes represent individual precursors from MS/MS spectra, and the edges indicate spectral similarity. Nodes are annotated as CP1 for intact C-peptide 1 and its truncated forms, whereas CP2 denotes intact C-peptide two and its corresponding truncated variants. Species that could not be identified are labeled as unknown (“UK”). The precursor *m/z* values, network labels, and shared fragment ions for the unidentified species are summarized in Table 1. Node color intensity increases with the number of connections, reflecting higher spectral similarity and network connectivity.

**Table 1:** Precursor *m/z* values, network annotations, edge connectivity, and shared fragment ions for unidentified species in the GNPS molecular network

Node label	Precursor *m/z* (charge)	Neutral mass (Da)	Edge connection	Common fragments
UK1	582.787 (−4)	2335	des-(26–31)-C-peptide 2	c_3_^−^, b_4_^−^, b_14_-H_2_O^2−^
UK2	602.784 (−4)	2415		c_3_^−^, b_4_^−^, c_19_^3-^, c_19_-H_2_O^4−^, b_14_-H_2_O^2-^
UK3	564.776 (−4)	2264		c_2_-H_2_O^−^, c_3_^−^, b_4_^−^, b_14_-H_2_O^2-^
UK4	611.057 (−4)	2448		c_3_^−^, c_19_^3−^, b_14_-H_2_O^2-^
UK5	536.756 (−4)	2151	des-(31)-C-peptide 2	c_2_-H_2_O^−^, b_4_^−^, b_14_-H_2_O^2−^
UK6	624.536 (−4)	2502		c_2_-H_2_O^−^, c_3_^−^, b_4_^-^, c_19_^3−^, b_14_-H_2_O^2−^

The molecular network reveals clustering of intact and truncated C-peptide two species, which are interconnected by multiple edges, reflecting high similarity of their MS/MS fragmentation patterns. A similar clustering pattern is observed for intact and truncated C-peptide 1 species. In addition, cross-connectivity is observed between the C-peptide 1 and C-peptide 2 clusters, including connections between intact C-peptides and between des-(24–29)-C-peptide 1 and des-(26–31)-C-peptide 2 and des-(24–31)-C-peptide 2. These connections arise from shared fragment ions observed in their MS/MS spectra, such as b_2_^−^, c_3_^−^, and b_4_^−^ ions (Figs. 3, supplemental Figs. S7, S14, and S16).

Further examination of the network indicates that six unidentified species (UK1–UK6) are directly connected to identified C-peptides. Specifically, UK1–UK4 are connected to des-(26–31)-C-peptide 2, while UK5 and UK6 are connected to des-(31)-C-peptide 2. Shared fragment ions between these unidentified species and their connected C-peptides are summarized in Table 1, with representative compared MS/MS spectra shown in supplemental Figs. S19–S21. We conclude that UK1–UK6 likely correspond to modified forms of truncated C-peptides.

### Spatial Distributions of the Intact and Truncated C-Peptides

**Fig. 5:**
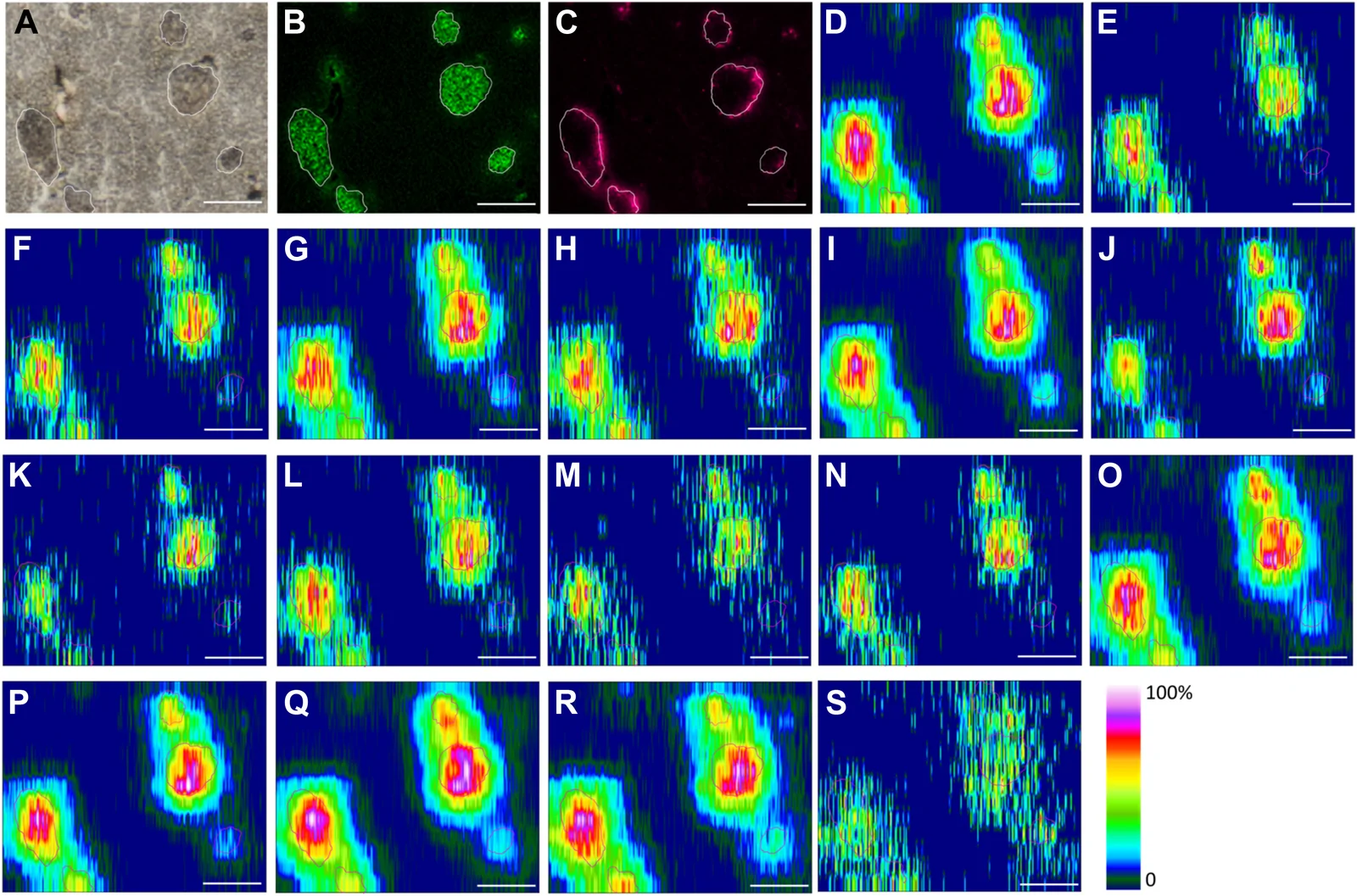
**Ion images of the identified C-peptides showing their spatial distribution in and around islets.** (*A*) Wide-field optical image of the analyzed region of the pancreatic tissue section. IF image of (*B*) INS and (*C*) glucagon on the adjacent section. Ion images of peptides normalized to TIC: (*D*) *m/z* 623.1063^5−^, 3121 Da, C-peptide 1, (*E*) *m/z* 559.0068^4−^, 2240 Da, des-(22-29)-C-peptide 1, (*F*) *m/z* 469.6200^5−^, 2353 Da, des-(23-29)-C-peptide 1, (*G*) *m/z* 483.8282^5−^, 2424 Da, des-(24-29)-C-peptide 1, (*H*) *m/z* 633.3064^4−^, 2537 Da, des-(25-29)-C-peptide 1, (*I*) *m/z* 625.5064^5−^, 3133 Da, C-peptide 2, (*J*) *m/z* 374.5096^3−^, 1126 Da, des-(11-31)-C-peptide 2, (*K*) *m/z* 341.1621^4−^, 1369 Da, des-(13-31)-C-peptide 2, (*L*) *m/z* 606.2909^3−^, 1822 Da, des-(20-31)-C-peptide 2, (*M*) *m/z* 511.4924^4−^, 2050 Da, des-(22-31)-C-peptide 2, (*N*) *m/z* 543.7549^4−^, 2179 Da, des-(23-31)-C-peptide 2, (*O*) *m/z* 569.0170^4−^, 2280 Da, des-(24-31)-C-peptide 2, (*P*) *m/z* 597.2876^4−^, 2393 Da, des-(25-31)-C-peptide 2, (*Q*) *m/z* 615.0473^4−^, 2464 Da, des-(26-31)-C-peptide 2, (*R*) *m/z* 643.3176^4−^, 2577 Da, des-(27-31)-C-peptide 2, (*S*) *m/z* 599.6972^5−^, 3004 Da, des-(31)-C-peptide 2. Scale bar: 250 μm. *Pink* trace delineates the islets outline drawn based on (*B*) INS IF image.

Ion images of all identified peptides in negative ion mode are shown in Figure 5 with additional ion images from replicate datasets provided in supplemental Figs. S22 and S23. The outlines of the islets were determined based on IF images of insulin and glucagon (Fig. 5, *B* and *C*) and are overlaid on each image in Figure 5. Signals of both intact C-peptide isoforms and their truncated forms are enhanced in the islets, with gradients extending into acinar cells. Notably, different C-peptides show different chemical gradients between the endocrine and acinar tissue. For example, some peptide signals (Fig. 5, *E*, *F*, *J*, *K*, *M*, and *N*) exhibit a steep decline (steep chemical gradient) at islet boundaries, indicating their tight localization to endocrine cells. Meanwhile, several other peptides (Fig. 5, *D*, *G–I*, *L*, *O–S*) show shallower chemical gradients that extend farther into the acinar tissue.

### Spatial Distributions of Other Multiply Charged Species

**Fig. 6:**
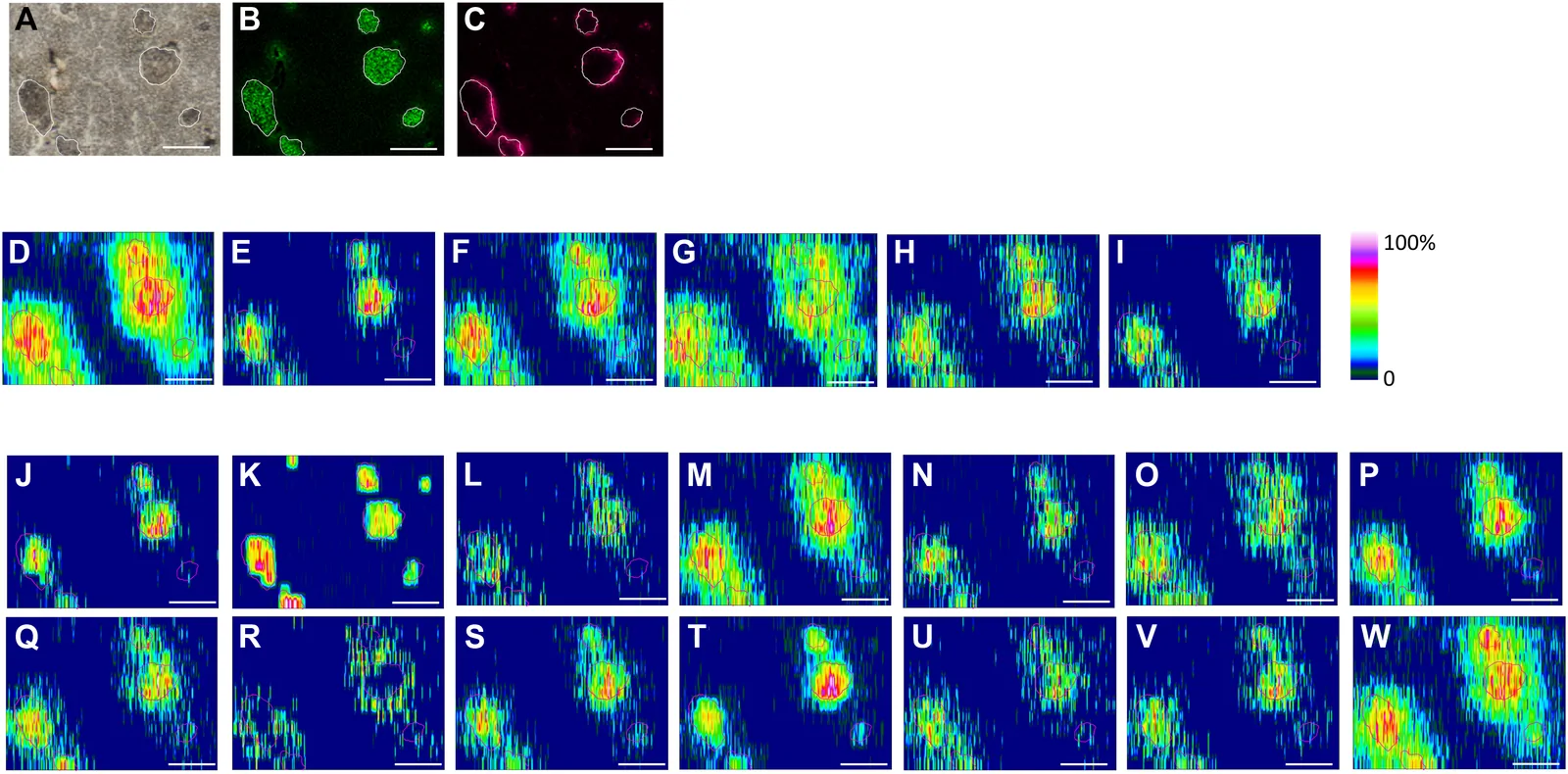
**Ion images of several unidentified peptides showing the spatial distribution in and around islets.***Top panel*: (*A*) Wide-field optical image of the analyzed region of the pancreatic tissue section. IF image of (*B*) INS and (*C*) glucagon on the adjacent section. Ion images of unidentified peptides normalized to TIC: *Middle panel* (*D*) UK1, *m/z* 582.7870^4−^, 2335 Da, (*E*) UK2, *m/z* 602.7839^4−^, 2415 Da, (*F*) UK3, *m/z* 565.0266^4−^, 2264 Da, (*G*) UK4, *m/z* 611.0578^4−^, 2448 Da, (*H*) UK5, *m/z* 536.7561^4−^, 2151 Da, (*I*) UK6, *m/z* 624.5357^4−^, 2502 Da; *Bottom panel*: (*J*) *m/z* 676.3299^2−^, 1355 Da, (*K*) *m/z* 720.8988^2−^, 1444 Da, (*L*) *m/z* 436.7023^4−^, 1751 Da, (*M*) *m/z* 572.7758^4−^, 2295 Da, (*N*) *m/z* 574.5125^4−^, 2302 Da, (*O*) *m/z* 601.0472^4−^, 2408 Da, (*P*) *m/z* 620.5431^4−^, 2486 Da, (*Q*) *m/z* 648.8137^4−^, 2599 Da, (*R*) *m/z* 750.8808^4−^, 3007 Da, (*S*) *m/z* 629.9023^5−^, 3154 Da, (*T*) *m/z* 640.8114^2−^, 1284 Da, (*U*) *m/z* 639.6412^3−^, 1922 Da, (*V*) *m/z* 555.0167^4−^, 2224 Da, (*W*) *m/z* 526.7459^4−^, 2111 Da. Scale bar: 250 μm. *Pink* trace delineates the islets outline drawn based on (*B*) INS IF image.

The spatial distributions of unidentified peptides are shown in Figure 6 with additional ion images from replicate datasets provided in supplemental Figs. S24 and S25. Some of these species are tightly confined to endocrine regions, whereas others display broader distributions extending into peri-insular acinar tissue. To further assess the spatial characteristics of unidentified species associated with C-peptides, ion images of peptides that cluster with C-peptides in the molecular network (UK1–UK6) are shown in the middle panel of Figure 6. As discussed earlier, UK1–UK4 are structurally similar to des-(26–31)-C-peptide 2, while UK5 and UK6 are structurally similar to des-(31)-C-peptide 2. Spatially, UK1 (Fig. 6*D*), UK3 (Fig. 6*F*), UK4 (Fig. 6*G*), and UK5 (Fig. 6*H*) show signal spreading from endocrine regions into peri-insular acinar tissue, whereas UK2 (Fig. 6*E*) and UK6 (Fig. 6*I*) remain more tightly localized within islet interiors.

**Fig. 7:**
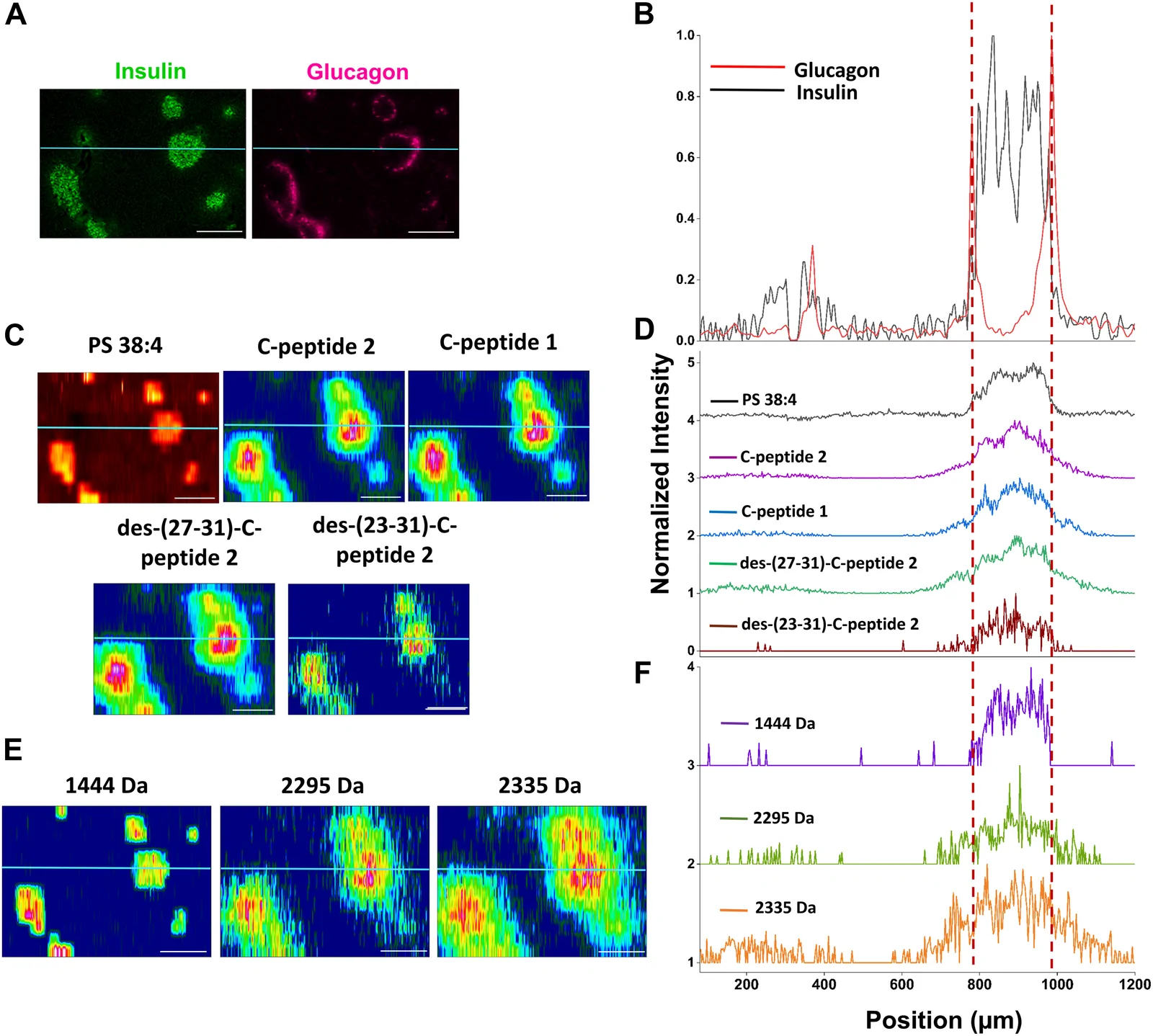
**Line profiles of selected peptides reveal sequence-dependent differences in chemical gradients.** (*A*) Immunofluorescence images of insulin and glucagon with the cyan line indicating the location of the extracted line profiles. (*B*) extracted line profiles of insulin and glucagon from the immunofluorescence images. *C*, ion images and (*D*) line profiles of PS(38:4), intact C-peptides, and truncated C-peptides with the cyan lines indicating the location of the extracted line profiles. *E*, ion images and (*F*) line profiles of selected unidentified species showing distinct spatial gradients. *Red dashed lines* indicate the approximate islet boundaries defined by the insulin/glucagon and PS(38:4) profiles. In panels (*B*), (*D*), and (*F*) line profiles were normalized to the maximum signal of the corresponding ion. Line profiles in panels *D* and *F* are vertically offset for clarity. Scale bar: 250 μm.

Ion images of unidentified peptides that do not participate in the molecular network are shown in the bottom panel of Figure 6. Several of these species, including peptides with intact masses of 1284 Da (Fig. 6*T*), 1355 Da (Fig. 6*J*), 1444 Da (Fig. 6*K*), 1751 Da (Fig. 6*L*), 1922 Da (Fig. 6*U*), 2224 Da (Fig. 6*V*), and 3154 Da (Fig. 6*S*) are also observed in higher abundance in the islets. Notably, the 1444 Da peptide (Fig. 6*K*) is exclusively localized to endocrine cells and exhibits the steepest spatial gradient at the islet boundary Fig. 7 suggesting its potential utility as an islet marker. In contrast, a 3007 Da peptide (Fig. 6*R*) is restricted to peri-insular acinar tissue and is absent from endocrine regions.

Line profiles of several peptides extracted from ion images are compared with line profiles of insulin and glucagon extracted from IF images in Figure 7, demonstrating differences in chemical gradients between endocrine and exocrine cells. Although we were unable to identify these peptides, their MS/MS spectra are provided in supplemental Figs. S26–S45 for reference.

## Discussion

Prior MS-based studies have provided important insights into pancreatic islet peptides, particularly those derived from insulin and proinsulin. Ramström *et al*. used LC-MS to analyze mouse islet extracts, reporting the presence of insulin 1/2, intact C-peptide 1 and C-peptide 2, two truncated C-peptides, glucagon, and glicentin-related pancreatic peptide (GRPP) (11). The mouse and human islet peptidome was further expanded by Galvin *et al*., who identified major endocrine peptides and processing products, including insulin/proinsulin-derived peptides, C-peptide, glucagon, islet amyloid polypeptides (IAPP), and somatostatin using bulk LC-MS analysis (38). More recently, Sisnande *et al*. employed positive-mode MALDI-MSI to map pancreatic hormone proteoforms, combining it with positive-mode LC-ESI-MS/MS of isolated islet extracts for cross-validation (13). Notably, twelve C-peptide species detected and identified in our study match C-peptides identified by Sisnande *et al*. using LC-ESI-MS/MS analysis of isolated islet extracts, providing independent validation of our findings.

While Jansson *et al*. demonstrated peptide heterogeneity at the single-islet-cell level, further emphasizing the importance of retaining cellular and spatial context (6), the present study achieves this directly *in situ*. By using negative-mode nano-DESI MSI combined with on-tissue MS/MS, we mapped and identified a family of intact and truncated C-peptides in untreated mouse pancreatic tissues alongside endogenous lipids and metabolites. This approach directly links peptide identities to islet-specific spatial localization, providing chemical and anatomical context across the tissue microenvironment without requiring complex sample processing. Although negative-mode LC- or CE-MS/MS of isolated islets could theoretically provide additional information, such workflows remain uncommon in islet peptidomics due to challenges associated with peptide identification in negative ion mode.

Nano-DESI MSI revealed a rich peptide population in mouse pancreas (Figs. 5 and 6), highlighting the power of this approach for studying their role in pancreatic biology. Manual annotation of MS/MS data allowed us to identify a family of C-peptides displaying distinct localization in and around the islets. Molecular networking analysis of MS/MS data revealed structural similarity within families of C-peptide 1 and C-peptide 2 and their truncated analogs, alongside extensive cross-connectivity between C-peptide 1 and C-peptide 2 species (Fig. 4). Although some peptide species remained unidentified, molecular networking identified several unknowns that are structurally related to the C-peptide family. Shared fragment ions (Table 1) between these unknowns and characterized C-peptides suggest they are additional truncated or modified C-peptide forms that remain to be fully characterized. By grouping species by MS/MS fragmentation similarity, molecular networking offers a systematic framework for interpreting complex peptide datasets even when complete structural information is lacking. Future advances in data-analysis methodologies, including improved de novo sequencing and complementary fragmentation methods, should enable unambiguous identification of these species.

### Spatial Mapping of Intact and Truncated C-Peptides Reveals Potential Endocrine-Exocrine Crosstalk

Both IF and ion images of several phospholipids and sphingomyelins that outline the pancreatic islets (Fig. 1) help contextualize the observed spatial distributions. A majority of C-peptides are expressed both in the endocrine tissue and acinar cells in the peri-insular regions (Fig. 5). Exocrine cells are located in close proximity to endocrine cells in the pancreas and likely influence their functional state (39, 40). Several studies highlight the importance of endocrine-exocrine crosstalk in β-cell proliferation. For example, exocrine enzymes such as elastase and carboxyl ester lipase affect endocrine cell function, proliferation, and longevity (41). Conversely, endocrine peptides support exocrine cell function via the insulo-acinar portal system (42). Endocrine hormones also promote (pancreatic polypeptide), inhibit (glucagon, somatostatin, and amylin), and regulate (insulin, ghrelin and cholecystokinin) exocrine enzyme secretion (42). Pathological perturbations within one pancreatic compartment can compromise the structural organization and functional capacity of the other, underscoring the regulatory interplay between endocrine and exocrine tissues (41). Because C-peptides are produced from proinsulin in β-cells, their shallow chemical gradients extending from islets into peri-insular exocrine tissue suggest transport of C-peptides to exocrine cells, likely through exocytosis (43), highlighting their potential role in endocrine-exocrine communication.

Proinsulin fragments, including B chain segments, truncated C-peptide segments, and hybrid insulin peptides (HIP) are autoantigens presented on MHC-II (major histocompatibility complex) on antigen-presenting cells and can be recognized by islet-infiltrating helper CD4+ T cells in type 1 diabetes (43, 44, 45, 46). MHC-II presentation of proinsulin-derived islet antigens can also engage regulatory T cells and, in some contexts, promote immune tolerance (47). The C19-A3 epitope, a proinsulin C-A chain junction peptide that includes the C-terminal half of C-peptide, has been tested as a tolerogenic immunotherapy and shown promise in preserving β-cell function in some studies (48, 49). The concentration gradients of autoangiogenic C-peptides we observe in healthy pancreatic tissue be relevant to these immune interactions. We hypothesize that the observed differences in chemical gradients of truncated C-peptides reflect differences in their functions.

### Islet-Associated Localization of Unidentified Peptides

Unidentified peptide species show distinct spatial distributions in pancreatic tissue (Fig. 6). Several peptides assigned to the C-peptide family using molecular networking display C-peptide-like localization patterns, with strong signals in the islet core and shallow gradients extending into peri-insular regions (Fig. 6, middle). Other non-networked peptides show similar spatial distributions. In contrast, the 1444 Da peptide is exclusively localized to endocrine cells, while the 3007 Da peptide is only observed in peri-insular acinar tissue, indicating compartment-specific molecular composition (Fig. 6, bottom).

The spatial gradients between endocrine and exocrine regions reveal region-dependent peptide distributions in pancreatic tissue. Although some peptides remain unidentified, the observed spatial patterns suggest these peptides may represent additional islet-associated species. Further structural characterization is needed to determine their identities and biological roles.

### Line Profiles of Identified and Unidentified Species

To evaluate whether the observed differences in peptide distributions are due to differences in absolute signals, we extracted line profiles of multiple species along the line shown in Figure 7 and compared them with line profiles of insulin and glucagon extracted from immunofluorescence images. The MSI-derived line profiles were TIC-normalized, normalized to the maximum abundance of each ion, and vertically offset to enable comparison of their spatial profiles independent of absolute abundance. The profile of PS(38:4), an islet-localized lipid marker, exhibits a steep decline at the islet boundary, closely following the endocrine region defined by insulin/glucagon staining. A similar steep decrease is observed for des-(23–31)-C-peptide, whereas other C-peptides shown in Panel D display broader spatial profiles that gradually extend into the surrounding acinar tissue. These analyte-specific line-profile shapes indicate that the observed differences in spatial gradients are not solely attributable to variations in peptide abundance or detection threshold but instead reflect distinct apparent distributions of C-peptide-related species across the endocrine–exocrine interface.

## Conclusion

This study leverages the sensitivity of nano-DESI MSI to identify and spatially map endogenous peptides, including previously unreported species, directly in mouse pancreatic tissue. These acidic peptides were observed as multiply deprotonated species exclusively in negative mode nano-DESI MSI. They were attributed to C-peptides, which are known to be derived from proinsulin within pancreatic islets. We obtained spatial maps of 36 peptide species in the pancreas. On-tissue MS/MS enabled confident identification of intact C-peptides 1 and 2 alongside fourteen truncated variants, revealing substantial peptide heterogeneity within pancreatic islets.

By integrating molecular networking with spatially resolved ion imaging, we established structural relationships among intact, truncated, and unidentified peptides based on shared MS/MS fragmentation features. Network-associated unknown species showed spatial distributions that closely paralleled those of identified C-peptides, while also revealing peptide-specific differences in endocrine confinement and endocrine-exocrine gradients across islet boundaries.

This study establishes an integrated framework combining nano-DESI-MSI, on-tissue MS/MS, and molecular networking for spatially resolved peptide discovery. The approach expands the catalog of pancreatic peptides and enables future studies of peptide processing in complex pancreatic tissues. Extending this platform to disease models and quantitative workflows will support systematic studies of intact and truncated peptides in pancreatic physiology and pathology.

## Data Availability

The raw nano-DESI mass spectrometry imaging and on-tissue MS/MS datasets supporting this study have been deposited in the MassIVE repository in vendor format under accession number MSV000102477. Processed ion images, C-peptide assignments, and associated analytical information are provided in the manuscript and supplemental data.

## Supplemental Data

This article contains supplemental data.

## Declaration of Generative AI and AI-Assisted Technologies in the Writing Process

Generative artificial intelligence tools were used only for language editing. All scientific content, data analysis, interpretation, and conclusions were generated and verified by the authors.

## Conflict of Interest

The authors declare that they have no conflicts of interest with the contents of this article.
