# Supplementary material for: Simultaneous Spatial Mapping and Identification of C-Peptides Alongside Lipids in Mouse Pancreatic Tissues with Nano-DESI Mass Spectrometry Imaging
Source: Mol Cell Proteomics. 2026 Jul 31;25(9):101632. doi: 10.1016/j.mcpro.2026.101632 (PMC13545366; doi:10.1016/j.mcpro.2026.101632)
Supplement: Supplementary file 1 [file mmc1.docx]

Supplemental Data for

Simultaneous Spatial Mapping and Identification of C-Peptides Alongside Lipids in Mouse Pancreatic Tissues with nano-DESI Mass Spectrometry Imaging

Manxi Yang,^a^^[1]^ Mushfeqa Iqfath,^a†^ Frederick Nguele Meke,^b^ Zihan Qu,^a,b^ Emerson L. Hernly,^a^ Xindi Tang, ^a^ Pei Su,^c^ Zhong-Yin Zhang,^a,b^ Julia Laskin^a^

^a^ Department of Chemistry, Purdue University, West Lafayette, IN, USA, 47907.

^b^ Borch Department of Medicinal Chemistry and Molecular Pharmacology, Purdue University, West Lafayette, IN, USA, 47907.

^c^ Department of Chemistry, Northwestern University, Evanston, IL, 60208.

**Corresponding Author:** Julia Laskin

William F. and Patty J. Miller Professor

Department of Chemistry, Purdue University

Running Title: Spatial Mapping of Truncated C-Peptides

Supplemental Table: **Table S1.** Summary of intact and truncated C-peptides identified in mouse pancreatic tissue, including peptide sequence, precursor charge state, observed m/z, neutral mass, and prior literature reports

**Supplemental Figures**

Fig. S1. Multimodal imaging of pancreatic tissue section workflow. The individual steps are described in detail in the Materials and Research Design section.

Fig. S2. Extracted line profiles of lipids and metabolites from ion images and extracted line profiles of insulin and glucagon from IF images.

(A) The ion images of lipids with the light blue trace marking the location of the extracted line profiles. Overlay of the line profiles of lipids detected in (B) positive ionization mode and (C) negative ionization mode. (D) The insulin and glucagon IF images with the light blue trace marking the location of (E) the extracted line profiles. Scale bar: 250 µm. The signal intensities were self-normalized

Fig. S3. Ion images of lipids and metabolites in mouse pancreas (replicate 2). (A) Brightfield optical image of the analyzed region of the pancreatic tissue section. IF image of (B) insulin and (C) glucagon on the adjacent section. Nano-DESI ion images of lipids and metabolites normalized to TIC with (D) species showing enhanced abundance and (E) species showing diminished abundance in the islets. Scale bar: 250 µm

Fig. S4. Ion images of lipids and metabolites in mouse pancreas (replicate 3). (A) Brightfield optical image of the analyzed region of the pancreatic tissue section. IF image of (B) insulin and (C) glucagon on the adjacent section. Nano-DESI ion images of lipids and metabolites normalized to TIC with (D) species showing enhanced abundance and (E) species showing diminished abundance in the islets. Scale bar: 250 µm

Fig. S5. MS/MS spectrum (top), zoomed-in MS/MS spectrum (middle) and the fragmentation map (bottom) of the -5 charge state of the 2240 Da des-(22-29)-C-peptide-1 at *m/z* 447.0035.

Fig. S6. MS/MS spectrum (top), zoomed-in MS/MS spectrum (middle) and the fragmentation map (bottom) of the -5 charge state of 2353 Da des-(23-29)-C-peptide-1 at *m/z* 469.62.

.

Fig. S7. MS/MS spectrum (top), zoomed-in MS/MS spectrum (middle) and the fragmentation map (bottom) of the -5 charge state of 2424 Da des-(24-29)-C-peptide-1 at *m/z* 483.8278.

Fig. S8. MS/MS spectrum (top), zoomed-in MS/MS spectrum (middle) and the fragmentation map (bottom) of the -5 charge state of 2537 Da des-(25-29)-C-peptide-1 at *m/z* 506.4447.

Fig. S9. MS/MS spectrum (top), zoomed-in MS/MS spectrum (middle) and the fragmentation map (bottom) of the -3 charge state of the 1126 Da des-(11-31)-C-peptide-2 at *m/z* 374.5096.

Fig. S10. MS/MS spectrum (top), zoomed-in MS/MS spectrum (middle) and the fragmentation map (bottom) of the -4 charge state of the 1368 Da des-(13-31)-C-peptide-2 at *m/z* 341.161.

Fig. S11. MS/MS spectrum (top), zoomed-in MS/MS spectrum (middle) and the fragmentation map (bottom) of the -3 charge state of the 1821 Da des-(20-31)-C-peptide-2 at *m/z* 606.291.

Fig. S12. MS/MS spectrum (top), zoomed-in MS/MS spectrum (middle) and the fragmentation map (bottom) of the -4 charge state of the 2050 Da des-(22-31)-C-peptide-2 at *m/z* 511.4920.

Fig. S13. MS/MS spectrum (top), zoomed-in MS/MS spectrum (middle) and the fragmentation map (bottom) of the -4 charge state of the 2179 Da des-(23-31)-C-peptide-2 at *m/z* 543.755.

Fig. S14. MS/MS spectrum (top), zoomed-in MS/MS spectrum (middle) and the fragmentation map (bottom) of the -5 charge state of the 2280 Da des-(24-31)-C-peptide-2 at *m/z* 455.012.

Fig. S15. MS/MS spectrum (top), zoomed-in MS/MS spectrum (middle) and the fragmentation map (bottom) of the -5 charge state of the 2393 Da des-(25-31)-C-peptide-2 at *m/z* 477.629.

Fig. S16. MS/MS spectrum (top), zoomed-in MS/MS spectrum (middle) and the fragmentation map (bottom) of the -5 charge state of the 2464 Da des-(26-31)-C-peptide-2 at *m/z* 491.8364.

Fig. S17. MS/MS spectrum (top), zoomed-in MS/MS spectrum (middle) and the fragmentation map (bottom) of the -5 charge state of the 2577 Da des-(27-31)-C-peptide-2. at *m/z* 514.4532.

Fig. S18. MS/MS spectrum (top), zoomed-in MS/MS spectrum (middle) and the fragmentation map (bottom) of the -5 charge state of the 3003 Da des-(31)-C-peptide-2 at *m/z* 599.697.

Fig. S19. The fragmentation spectrum (a) -4 charge state of species with mass 2335 Da at *m/z* 582.7869; (b) ) -4 charge state of species with mass 2415 Da at *m/z* 602.784; (c) -4 charge state of species with mass 2448 Da at *m/z* 611.057; (d) the -4 charge state of the 2464 Da Des-(26-31)-C-peptide 2 at *m/z* 615.047.

Fig. S20. The fragmentation spectrum (a) -4 charge state of species with mass 2151 Da at *m/z* 536.756 (b) -5 charge state of species with mass 2502 Da at *m/z* 624.536; (c) the -5 charge state of the 2464 Da Des-(31)-C-peptide 2 at *m/z* 599.898.

Fig. S21. The fragmentation spectrum (a) -4 charge state of species with mass 2264 Da at *m/z* 564.776; (b) -4 charge state of the 2464 Da Des-(26-31)-C-peptide 2 at *m/z* 615.047.

Fig. S22. Ion images of identified pancreatic peptides from replicate 2.

(A) Brightfield optical image of the analyzed region of the pancreatic tissue section. IF image of (B) INS and (C) glucagon on the adjacent section. Ion images of peptides normalized to TIC: (D) *m/z* 623.1063^5-^, 3121 Da, C-peptide 1, (E) *m/z* 559.0068^4-^, 2240 Da, des-(22-29)-C-peptide 1, (F) *m/z* 469.6200^5-^, 2353 Da, des-(23-29)-C-peptide 1, (G) *m/z* 483.8282^5-^, 2424 Da, des-(24-29)-C-peptide 1, (H) *m/z* 633.3064^4-^, 2537 Da, des-(25-29)-C-peptide 1, (I) *m/z* 625.5064^5-^, 3133 Da, C-peptide 2, (J) *m/z* 374.5096^3-^, 1126 Da, des-(11-31)-C-peptide 2, (K) *m/z* 341.1621^4-^, 1369 Da, des-(13-31)-C-peptide 2, (L) *m/z* 606.2909^3-^, 1822 Da, des-(20-31)-C-peptide 2, (M) *m/z* 511.4924^4-^, 2050 Da, des-(22-31)-C-peptide 2, (N) *m/z* 543.7549^4-^, 2179 Da, des-(23-31)-C-peptide 2, (O) *m/z* 569.0170^4-^, 2280 Da, des-(24-31)-C-peptide 2, (P) *m/z* 597.2876^4-^, 2393 Da, des-(25-31)-C-peptide 2, (Q) *m/z* 615.0473^4-^, 2464 Da, des-(26-31)-C-peptide 2, (R) *m/z* 643.3176^4-^, 2577 Da, des-(27-31)-C-peptide 2, (S) *m/z* 599.6972^5-^, 3004 Da, des-(31)-C-peptide 2. Scale bar: 250 µm.

Fig. S23. Ion images of identified pancreatic peptides from replicate 3.

(A) Brightfield optical image of the analyzed region of the pancreatic tissue section. IF image of (B) INS and (C) glucagon on the adjacent section. Ion images of peptides normalized to TIC: (D) *m/z* 623.1063^5-^, 3121 Da, C-peptide 1, (E) *m/z* 559.0068^4-^, 2240 Da, des-(22-29)-C-peptide 1, (F) *m/z* 469.6200^5-^, 2353 Da, des-(23-29)-C-peptide 1, (G) *m/z* 483.8282^5-^, 2424 Da, des-(24-29)-C-peptide 1, (H) *m/z* 633.3064^4-^, 2537 Da, des-(25-29)-C-peptide 1, (I) *m/z* 625.5064^5-^, 3133 Da, C-peptide 2, (J) *m/z* 374.5096^3-^, 1126 Da, des-(11-31)-C-peptide 2, (K) *m/z* 341.1621^4-^, 1369 Da, des-(13-31)-C-peptide 2, (L) *m/z* 606.2909^3-^, 1822 Da, des-(20-31)-C-peptide 2, (M) *m/z* 511.4924^4-^, 2050 Da, des-(22-31)-C-peptide 2, (N) *m/z* 543.7549^4-^, 2179 Da, des-(23-31)-C-peptide 2, (O) *m/z* 569.0170^4-^, 2280 Da, des-(24-31)-C-peptide 2, (P) *m/z* 597.2876^4-^, 2393 Da, des-(25-31)-C-peptide 2, (Q) *m/z* 615.0473^4-^, 2464 Da, des-(26-31)-C-peptide 2, (R) *m/z* 643.3176^4-^, 2577 Da, des-(27-31)-C-peptide 2, (S) *m/z* 599.6972^5-^, 3004 Da, des-(31)-C-peptide 2. Scale bar: 250 µm.

Fig. S24. Ion images of other unidentified species obtained from replicate 2.

(A) Brightfield optical image of the analyzed region of the pancreatic tissue section. IF image of (B) INS and (C) glucagon on the adjacent section. Ion images of other multimply charged species normalized to TIC: (D) *m/z* 640.8114^2-^, 1284 Da, (E) *m/z* 676.3299^2-^, 1355 Da, (F) *m/z* 720.8988^2-^, 1444 Da, (G) *m/z* 436.7023^4-^, 1751 Da, (H) *m/z* 639.6412^3-^, 1922 Da, (I) *m/z* 526.7459^4-^, 2111 Da, (J) *m/z* 536.7561^4-^, 2151 Da, UK5 (K) *m/z* 555.0167^4-^, 2224 Da, (L) *m/z* 565.0266^4-^, 2264 Da, UK3 (M) *m/z* 572.7758^4-^, 2295 Da, (N) *m/z* 574.5125^4-^, 2302 Da, (O) *m/z* 582.7870^4-^, 2335 Da, UK1 (P) *m/z* 601.0472^4-^, 2408 Da, (Q) *m/z* 602.7839^4-^, 2415 Da, UK2 (R) *m/z* 537.0578^4-^, 2448 Da, UK4 (S) *m/z* 620.5431^4-^, 2486 Da, (T) *m/z* 624.5357^4-^, 2502 Da, UK6 (U) *m/z* 648.8137^4-^, 2599 Da, (V) *m/z* 750.8808^4-^, 3007 Da, (W) *m/z* 629.9023^5-^, 3154 Da. Scale bar: 250 µm.

Fig. S25. Ion images of other unidentified species obtained from replicate 3.

(A) Brightfield optical image of the analyzed region of the pancreatic tissue section. IF image of (B) INS and (C) glucagon on the adjacent section. Ion images of other multimply charged species normalized to TIC: (D) *m/z* 640.8114^2-^, 1284 Da, (E) *m/z* 676.3299^2-^, 1355 Da, (F) *m/z* 720.8988^2-^, 1444 Da, (G) *m/z* 436.7023^4-^, 1751 Da, (H) *m/z* 639.6412^3-^, 1922 Da, (I) *m/z* 526.7459^4-^, 2111 Da, (J) *m/z* 536.7561^4-^, 2151 Da, UK5 (K) *m/z* 555.0167^4-^, 2224 Da, (L) *m/z* 565.0266^4-^, 2264 Da, UK3 (M) *m/z* 572.7758^4-^, 2295 Da, (N) *m/z* 574.5125^4-^, 2302 Da, (O) *m/z* 582.7870^4-^, 2335 Da, UK1 (P) *m/z* 601.0472^4-^, 2408 Da, (Q) *m/z* 602.7839^4-^, 2415 Da, UK2 (R) *m/z* 537.0578^4-^, 2448 Da, UK4 (S) *m/z* 620.5431^4-^, 2486 Da, (T) *m/z* 624.5357^4-^, 2502 Da, UK6 (U) *m/z* 648.8137^4-^, 2599 Da, (V) *m/z* 750.8808^4-^, 3007 Da, (W) *m/z* 629.9023^5-^, 3154 Da. Scale bar: 250 µm.

Fig. S26. MS/MS spectrum (top) and zoomed-in MS/MS spectrum (bottom) of the -2 charge state of the 1284 Da at *m/z* 640.8114.

Fig. S27. MS/MS spectrum (top) and zoomed-in MS/MS spectrum (bottom) of the -2 charge state of the 1355 Da at *m/z* 676.3299.

Fig. S28. MS/MS spectrum (top) and zoomed-in MS/MS spectrum (bottom) of the -2 charge state of the 1444 Da at *m/z* 720.8988.

Fig. S29. MS/MS spectrum (top) and zoomed-in MS/MS spectrum (bottom) of the -4 charge state of the 1751 Da at *m/z* 436.7023.

Fig. S30. MS/MS spectrum (top) and zoomed-in MS/MS spectrum (bottom) of the -3 charge state of the 1922 Da at *m/z* 639.6412.

Fig. S31. MS/MS spectrum (top) and zoomed-in MS/MS spectrum (bottom) of the -4 charge state of the 2111 Da at *m/z* 526.7459.

Fig. S32. MS/MS spectrum (top) and zoomed-in MS/MS spectrum (bottom) of the -4 charge state of the 2151 Da at *m/z* 536.7561.

Fig. S33. MS/MS spectrum (top) and zoomed-in MS/MS spectrum (bottom) of the -4 charge state of the 2224 Da at *m/z* 555.0167.

Fig. S34. MS/MS spectrum (top) and zoomed-in MS/MS spectrum (bottom) of the -4 charge state of the 2264 Da at *m/z* 565.0266.

Fig. S35. MS/MS spectrum (top) and zoomed-in MS/MS spectrum (bottom) of the -4 charge state of the 2295 Da at *m/z* 572.7758.

Fig. S36. MS/MS spectrum (top) and zoomed-in MS/MS spectrum (bottom) of the -4 charge state of the 2302 Da at *m/z* 574.5125.

Fig. S37. MS/MS spectrum (top) and zoomed-in MS/MS spectrum (bottom) of the -4 charge state of the 2335 Da at *m/z* 582.7870.

Fig. S38. MS/MS spectrum (top) and zoomed-in MS/MS spectrum (bottom) of the -4 charge state of the 2408 Da at *m/z* 601.0472.

Fig. S39. MS/MS spectrum (top) and zoomed-in MS/MS spectrum (bottom) of the -4 charge state of the 2415 Da at *m/z* 602.7839.

ss

Fig. S40. MS/MS spectrum (top) and zoomed-in MS/MS spectrum (bottom) of the -4 charge state of the 2448 Da at *m/z* 611.0578.

Fig. S41. MS/MS spectrum (top) and zoomed-in MS/MS spectrum (bottom) of the -4 charge state of the 2486 Da at *m/z* 620.5431.

Fig. S42. MS/MS spectrum (top) and zoomed-in MS/MS spectrum (bottom) of the -4 charge state of the 2502 Da at *m/z* 624.5357.

Fig. S43. MS/MS spectrum (top) and zoomed-in MS/MS spectrum (bottom) of the -4 charge state of the 2599 Da at *m/z* 648.8137.

Fig. S44. MS/MS spectrum (top) and zoomed-in MS/MS spectrum (bottom) of the -4 charge state of the 3007 Da at *m/z* 750.8808.

Fig. S45. MS/MS spectrum (top) and zoomed-in MS/MS spectrum (bottom) of the -5 charge state of the 3154 Da at *m/z* 629.9023.

**Table S1.** Summary of intact and truncated C-peptides identified in mouse pancreatic tissue, including peptide sequence, precursor charge state, observed m/z, neutral mass, and prior literature reports.^a^

| **Peptide name** | **Observed m/z** | **Charge** | **Mass (Da)** | **Sequence** | **Literature** | |
| --- | --- | --- | --- | --- | --- | --- |
| Intact C-peptide 1 | 623.106 | -5 | 3121 | EVEDPQVEQLELGGSPGDLQTLALEVARQ | | Ref.11, Ref. 13 |
| des-(25-29)-C-peptide 1 | 506.4447 | -5 | 2537 | EVEDPQVEQLELGGSPGDLQTLAL | | Ref.11, Ref. 13 |
| des-(24-29)-C-peptide 1 | 483.8278 | -5 | 2424 | EVEDPQVEQLELGGSPGDLQTLA | | Ref. 13 |
| des-(23-29)-C-peptide 1 | 469.6200 | -5 | 2353 | EVEDPQVEQLELGGSPGDLQTL | | Ref. 13 |
| des-(22-29)-C-peptide 1 | 447.0035 | -5 | 2240 | EVEDPQVEQLELGGSPGDLQT | | Ref. 13 |
| Intact C-peptide 2 | 625.5060 | -5 | 3133 | EVEDPQVAQLELGGGPGAGDLQTLALEVAQQ | | Ref.11, Ref. 13 |
| des-(31)-C-peptide 2 | 599.6970 | -5 | 3004 | EVEDPQVAQLELGGGPGAGDLQTLALEVAQ | | Present work |
| des-(27-31)-C-peptide 2 | 514.4532 | -5 | 2577 | EVEDPQVAQLELGGGPGAGDLQTLAL | | Ref.11, Ref. 13 |
| des-(26-31)-C-peptide 2 | 491.8364 | -5 | 2464 | EVEDPQVAQLELGGGPGAGDLQTLA | | Ref. 13 |
| des-(25-31)-C-peptide 2 | 477.6290 | -5 | 2393 | EVEDPQVAQLELGGGPGAGDLQTL | | Ref. 13 |
| des-(24-31)-C-peptide 2 | 455.0120 | -5 | 2280 | EVEDPQVAQLELGGGPGAGDLQT | | Ref. 13 |
| des-(23-31)-C-peptide 2 | 543.7550 | -4 | 2179 | EVEDPQVAQLELGGGPGAGDLQ | | Ref. 13 |
| des-(22-31)-C-peptide 2 | 511.4920 | -4 | 2050 | EVEDPQVAQLELGGGPGAGDL | | Ref. 13 |
| des-(20-31)-C-peptide 2 | 606.2910 | -3 | 1822 | EVEDPQVAQLELGGGPGAG | | Present work |
| des-(13-31)-C-peptide 2 | 341.1610 | -4 | 1369 | EVEDPQVAQLEL | | Present work |
| des-(11-31)-C-peptide 2 | 374.5096 | -3 | 1126 | EVEDPQVAQL | | Present work |

^a^Listed references correspond to citations in the main manuscript.

1. Contributed equally to this work
